# Leaf identification using radial basis function neural networks and SSA based support vector machine

**DOI:** 10.1371/journal.pone.0237645

**Published:** 2020-08-19

**Authors:** Ali Ahmed, Sherif E. Hussein

**Affiliations:** 1 IT Department, Faculty of Computers and Information, Menoufia University, Menoufia, Egypt; 2 Computer Engineering and Systems Department, Faculty of Engineering, Mansoura University, Mansoura, Egypt; Hefei University of Technology, CHINA

## Abstract

In this research, an efficient scheme to identify leaf types is proposed. In that scheme, the leaf boundary points are fitted in a continuous contour using Radial Basis Function Neural Networks (RBFNN) to calculate the centroid of the leaf shape. Afterwards, the distances between predetermined points and the centroid were computed and normalized. In addition, the time complexity of the features’ extraction algorithm was calculated. The merit of this scheme is objects’ independence to translation, rotation and scaling. Moreover, different classification techniques were evaluated against the leaf shape features. Those techniques included two of the most commonly used classification methods; RBFNN and SVM that were evaluated and compared with other researches that used complex features extraction algorithms with much higher dimensionality. Furthermore, a third classification method with an optimization technique for the SVM using Salp Swarm Algorithm (SSA) was utilized showing a significant improvement over RBFNN and SVM.

## I. Introduction

Nowadays, plants play an essential role in our life. They are beneficial for human food, medicine and industry. The identification of each species is a difficult task because of the existence of numerous plant species and the similarity between them. Automation and identification of plant species are extremely important for scientists interested in many fields including agriculture and botany.

The identification and recognition of plant species have been carried out by researchers for many years utilizing different plant features such as leaf shape, texture, and color. Different plant species have different features that provide beneficial information for researchers. One category of these features, commonly used in literature, is called histograms of oriented gradients (HOGs) that are applied in many visual object recognition applications [1]. While other research directions used parse representation of leaf tooth features for automatic pieces identification that include the number, rate, sharpness, and obliqueness concatenated to form a feature vector [2].

In another investigation, several geometric features (12 morphological features) were defined in [3] and divided into two different categories, namely general and domain-related visual features. A research by Yanikoglu et al. has utilized a general category of features that consists of color, texture, and shape features [4]. In addition, Jyotismita et al, characterized and recognized plant’s leaves by extracting both texture-based and shape-based features [5]. They applied Gabor filter and Gray-Level Co-occurrence Matrix to model leaf’s texture and calculate a set of Curvelet transform coefficients and invariant moments to represent the leaf’s shape. Another research direction used contour-based features such as centroid contour distance (CCD) and angle code (AC) that are considered the most popular features for many researchers [6]. The work presented in [7] extracted fifteen features from plant’s leaf identification. Among these features, eight geometric metrics such as rectangularity, circularity, eccentricity features together with moment invariant features. Moreover, an image-based description and measurement of the leaf’s teeth are used in [8].

In literature, various approaches for plant species identification and recognition are proposed by researchers. A deep learning scheme for the quantification and discrimination of plant leaf was presented in [9]. In their work, Convolutional Neural Networks (CNN) were used for learning useful leaf features that can be extracted directly from a dataset. Other researchers used a neuro-fuzzy controller (NFC) and a feed-forward back-propagation Multi-Layered Perceptron (MLP) to differentiate between thirty-one leaves’ classes [5]. Another type of classifiers, the Support Vector Machine (SVM) was trained with a portion of the training data using a subset of features which included color, shape, and texture features [4]. In a research presented in [10], a classification using k-Nearest Neighbor classifier was implemented and tested on 640 leaves belonging to 32 different species of plants based on a feature extraction stage. To distinguish leaf margins that cannot be easily differentiated by most of the commonly used geometric features, linear discriminant classifier (LDC) was utilized for leaves classification [11]. While another classification method called Moving Center Hypersphere (MCH) classifier was efficiently used to address high-dimensional leaf’s features [12]. In addition, the study presented in [13] used SVM for segmenting and classifying legume leaves based on the analysis of their veins. Tan et al. used a classifier, namely CNN for plant identification from leaf vein features [14]. They used the CNN classifier to discriminate between three types of legume species named white, red, and soybean. Moreover, a simple nearest neighbor classifier was used to classify plant leaves using texture features extracted by a modified local binary patterns (MLBP) [15]. Also, a simple linear discriminant (LDA) analysis and SVM were used in [16] to classify plant leaves. Table 1 summaries the related studies which are beneficial to compare the different identification techniques’ performance and their corresponding features.

**Table 1:** Summarization of related literature.

Reference	Year	List of Leaf features	Classifier	Classification Accuracy
Ref. [1]	2015	Histograms of Oriented Gradients (HOGs)	Probabilistic Neural Network (PNN)	86.07%
Ref. [2]	2015	Geometric features	A sparse representation-based classifier	76. 4%
Ref. [4]	2014	Shape, texture and colour features	Support Vector Machine (SVM)	80.69%
Ref. [5]	2015	Texture and shape features	A Neuro-Fuzzy Controller (NFC) and neural networks	67.7%.
Ref. [9]	2017	Shape, texture, and venation	Convolutional Neural Networks (CNN)	96.3%
Ref. [10]	2015	Global features	k-Nearest neighbour classifier (KNN)	83.5%
Ref. [11]	2015	Moments and shape	Linear Discriminant Classifier (LD)	94%
Ref. [13]	2019	Morphological features	Support Vector Machine (SVM)	88.33%
Ref. [14]	2018	Morphological features	Convolutional Neural Networks (CNN)	94.88%
Ref. [15]	2016	Texture	k-Nearest neighbour classifier (KNN)	90.62%
Ref. [16]	2014	geometry features	Support Vector Machine (SVM)	87.40%

In this research, two of the most successfully used classification techniques were presented and evaluated against the leaf shape features, namely RBFNN and SVM. Then, an optimization technique for the SVM using a Salp Swarm Algorithm (SSA) was proposed and compared with RBFNN and SVM. This research is organized as follows: Section two discusses in detail the different classification techniques used in this research; Section three presents the proposed leaf features extraction, Section four shows the results and discussion of the proposed work, and finally, Section five gives the conclusions for the proposed study and the suggestion for future research.

## II. Methods

The introduced feature extraction algorithm in this research uses the leaf shape to identify the geometrical features. Furthermore, the proposed approach will be assessed using three classification methods and compared with the performance of other techniques found in recent literature that identify the different plant species. The utilized classification techniques are discussed in the following subsections:

### A) RBFNN classification

Artificial neural network (ANN) can be incorporated in plenty of applications, including curve fitting, regression, and classification. Curve fitting is used to get the values for a set of parameters through which a given set of explanatory variables can depict another variable such as equation estimation using empirical methods. Regression, on the other hand, is unambiguously defined from the statistics perspective. In a sense, regression is not a mathematical concept; it's a statistical concept, while curve fitting is a mathematical concept. Regression is curve fitting under context: and the goal is to understand how one variable depends on another variable.

Radial basis function neural network (RBFNN) is the most commonly used approach for many research fields such as object classification, linear regression, curve fitting, and discrete-based data clustering [17]. It utilizes radial basis function as its activation function as in [18, 19]. RBFNN framework consists of three layers; input layer, hidden layer, and output layer. The first layer of RBFNN is the input vector to each unit in the next hidden layer. RBF activates each unit in the hidden layer. Then, a linear combination of the activations using each unit of the hidden layer is used to create a classified output. The output layer mainly depends on the activation function used and their weights combined with the links between both the hidden and the output layers. RBFNNs were used for both the feature extraction stage as well as the classification of the different leaf's types.

**Fig 1:**
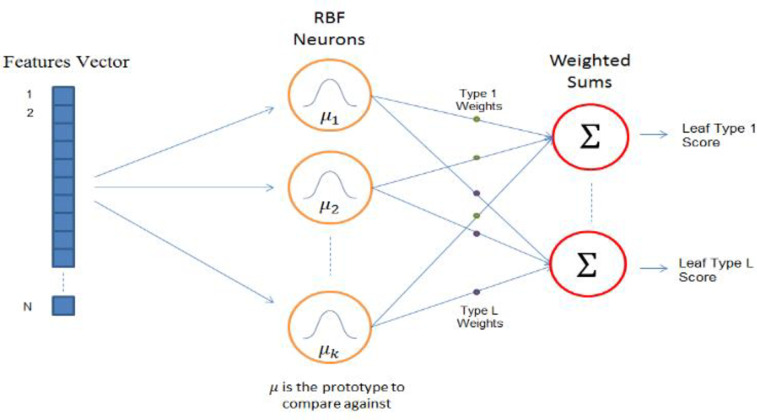
The RBFNN as a classifier.

For the RBFNN as a classifier the input vector of the RBFNN architecture is an n-dimensional vector that represented the extracted features. Each neuron in the hidden layer stores one of the training set vectors and computes the similarity between the input features vector and the corresponding prototype vector and according to this comparison, the output layers give a value between 0 and 1. If the output unit of a certain RBF neuron is close to 1, then the input is equal to the prototype. If the difference between the input unit and the prototype increases, the output unit value falls towards zero exponentially. The shape of the RBF response of neurons takes a bell-shaped curve and the value of neuron's response value is the activation of the neuron. RBF output layer consists of different nodes; each node represents a class and computes a value for its corresponding class. The classification decision is done by assigning the input vector to the class that achieves the highest score as shown in Fig 1.

There are different similarity functions; Gaussian function is considered the most popular one. The following equation is a one-dimensional Gaussian function.

$$G(x)=\frac{1}{\sigma \sqrt{2\pi }}e^{\frac{{\left(x-\mu \right)}^{2}}{2\sigma^{2}}}$$ (1)

where *x* in Eq (1) is the input vector, *μ* is the computed mean value, and *σ* represent the standard-deviation. The activation function of RBF neuron is different from Gaussian function and is written as:

$$\varphi (x)=e^{-\beta {∥x-\mu ∥}^{2}}$$ (2)

this function is the prototype vector that represent the center of the bell-shaped curve.

### B) Support vector machine classification

SVM method is considered one of the commonly used machine learning methods for classification and regression problems and was first introduced by Drucker [20]. The SVM classifier is used to classify the unknown sample by building the classification model from the training data. The classifier has two parameters called penalty and kernel parameters. These parameters have major effect on SVM performance [21]. The first one plays an important role as a tradeoff between training data error minimization and the maximization of the classification margin. The other is used to determine the nonlinear mapping between both the input and the high-dimensional feature space. Consequently, the way to choose the values of the above-mention parameters affects the performance of SVM [22, 23].

The mis-classification might result in because of non-separable data. So, the linear SVM constraints should be slowed down and that can be done by adding a slack variable *ξ*_*i*_ as shown in the following equation (Eq (3)). The slack variable *ξ*_*i*_ represents the distance between each training sample and the corresponding margin which should be minimized.

wTXi+B≥+1−ξiforYi=+1wTXi+B≤−1+ξiforYi=−1 (3)

These equations can be unified to one equation as follows:

$${Y_{i}(w}^{T}X_{i}+B)-1+\xi_{i}\geq 0$$ (4)

where the slack variable *ξ*_*i*_≥0

If 0 ≤ 0 *ξ*_*i*_ ≤ 1, then the sample is correctly classified as it lies between the classification margin and the correct side of the hyper plane. On the other hand, if *ξ*_*i*_ > 1, then, Y_i_(w^T^X_i_+B)≥1−ξ_i_; therefore, the function (w^T^X_i_+B) and the label (*Y*_*i*_) have different sign and accordingly, the sample data *X*_*i*_ is mis-classified.

After adding the variable *ξ*_*i*_ and the penalty parameter *C* to the objective function of SVM, we obtain the following equation:

min12∥w∥2+C∑i=1Nξis.t.yi(wTXi+B)−1+ξi≥0∀i=1,2,…,N (5)

By formalizing Eq (5) using Lagrange formula, it will appear as follows:

$$L_{P}=\frac{1}{2}{∥w∥}^{2}+C\sum_{i=1}^{N}\xi_{i}-\sum_{i=1}^{N}\alpha_{i}[{Y_{i}(w}^{T}X_{i}+B)-1+\xi_{i}]$$ (6)

where *α*_*i*_ in the equation is greater than zero.

After differentiating Lagrange formula *L*_*P*_ with respect to the three variables w, *B* and *ξ*_*i*_ we obtain the following equation after setting the result to zero:

$$\frac{\partial L_{P}}{\partial \xi_{i}}=0⟹C=\alpha_{i}+\xi_{i}$$ (7)

It is clear from the above equation that *α*_*i*_ determines the upper bound of the variable *C*. In addition, the support vectors (SVs) with the variable *α*_*i*_ = *C* can lie inside or outside the margin boundary.

In case the sample is non-linearly separable data, SVM maps it to a linearly separable, as a higher-dimensional data, using the kernel function Q. According to Eq (8), this kernel is represented as a dot product of the nonlinear functions. The objective function of SVM is defined by Eq (9).

$$K(X_{i},X_{j})={Q(X_{i})}^{T}Q(X_{j})$$ (8)

min12∥w∥2+C∑i=1Nξis.t.Yi(wTQ(Xi)+B)−1+ξi≥0∀i=1,2,…,N (9)

In literature, there are several kernel functions used for mapping the data. Some of these are the linear kernel function defined as K(X_I_,X_j_) = 〈X_I_,X_j_〉 and RBF is defined as ${K(X_{i},X_{j})=e}^{\frac{-{\left\|X_{i}-X_{j}\right\|}^{2}}{2\sigma^{2}}}$.

### C) Salp swarm algorithm

Salp Swarm Algorithm (SSA) is a random population-based algorithm introduced by [24] and recently used by [25, 26]. The idea behind SSA is to simulate the swarming behavior of salps when foraging in oceans. They usually shape a swarm as a salp chain when foraging. In the proposed SSA, the leader salp is the front of the chain and the followers are the remaining of salps chain. According to the implementation of other swarm-based techniques, the salps locations in the chain are represented by a two-dimensional matrix. In addition, the target of swarm is assumed to be the food source called *F*. The following is the mathematical model of Salp swarm algorithm:

In SSA implementation, the following proposed equation is used to update the leader’s position as follows:

$$p_{j}^{1}=\left\{ \begin{array}{l} F_{j}+s_{1}((up{pb}_{j}-l{owb}_{j})s_{2}+lowb_{j})\;s_{3}\geq 0.5\\ F_{j}-s_{1}((u{ppb}_{j}-lowb_{j})s_{2}+l{owb}_{j})\;s_{3}<0.5 \end{array}\right.$$ (10)

where $p_{j}^{1}$ is defined as the first leader’s position in jth dimension search space and *F*_*j*_ is the food source position in jth dimension, *uppb*_*j*_is the upper-bound and *lowb*_*j*_ is the lower-bound of jth dimension respectively, the two parameters *s*_2_, and *s*_3_ are random numbers. As seen from Eq (10), the leader’s position of salp chain is updated according to the food source position. The first parameter *s*_1_ has its importance in SSA because of its role of balancing between exploration and exploitation as follows:

$$s_{1}=2e^{-{\left(\frac{4L_{c}}{L_{M}}\right)}^{2}}$$ (11)

where the parameters *L*_*c*_ and *L*_*M*_ in Eq (11) are defined as the current and the maximum numbers of iterations, respectively. The parameters s_2_ and s_3_ are uniformly generated from the interval [0, 1].

By increasing iteration count, s_1_ parameter decreases. That, in turn, can put more emphasize on the diversification on initial stages and put more emphasize on the intensification tendency at the last steps of optimization. The location of followers is adjusted by the following equation [24]:

$$p_{j}^{i}=\frac{1}{2}(p_{j}^{i}+p_{j}^{i-1})$$ (12)

In this equation, *i*≥2 and the position $p_{j}^{i}$ represent the i^th^ follower’s position in j^th^ dimension space. The following algorithm is the pseudo code of SSA.

**Algorithm 1.** Pseudo code for the SSA.

**Initialize** p_i_ (i = 1,2,…,n) the position of salp′s population and consider uppb and lowb

**While** (the termination criteria are not met)

                 **Compute** all search agent’s fitness (salps)

                 F is the food source (the best search agent)

                 **Update** s_1_ according to Eq (11)

                 For each salp’s position (p_i_)

                                  If (i equal to 1)

                                  **Update** the leading salp’s location using Eq (10)

                                  else

                                  **Update** the follower salp’s location using Eq (12)

                                  end

                 end

                 **modify** the salps based on uppb and lowb

end

return F

### D) The SSA-SVM classifier

In this sub-section, the proposed SSA-SVM model in the framework is described to compute the optimal SVM parameters as follows:

- *Initialization*: the following parameters of SSA were initialized (salps number, maximum iterations, salp’s position and velocity).
- *Training*: in the SSA-SVM model, the SSA inputs the SVM classifier by the two values, penalty parameter C and RBF kernel parameter, to train SVM. Therefore, each salp position is represented a combination of two parameters (C and kernel parameters) in two-dimensional search space.
- *Testing*: the testing samples is used to compute the mis-classification (error rate) of each position dividing the number of mis-classified samples (N_err_) by the total number of testing samples (N_total_) according to Eq (13). When the values of the two above-mentioned parameters at certain position p_i_∈R^2^ reached the minimum error rate, the optimal solution is located at p_i_∈R^2^,

$$Minimize:\;F=\frac{N_{err}}{N_{total}}$$ (13)

- *Termination*: When the end condition is reached, the process terminated; if not, the next generation operation proceeds. In our model, the SSA is ended when the operation reached the max iterations, or the obtained best solution is not changed within a certain number of iterations. In this case, the salp positions are then updated according to (Eqs 10 and 12).

## III. Leaf features extraction

**Fig 2:**
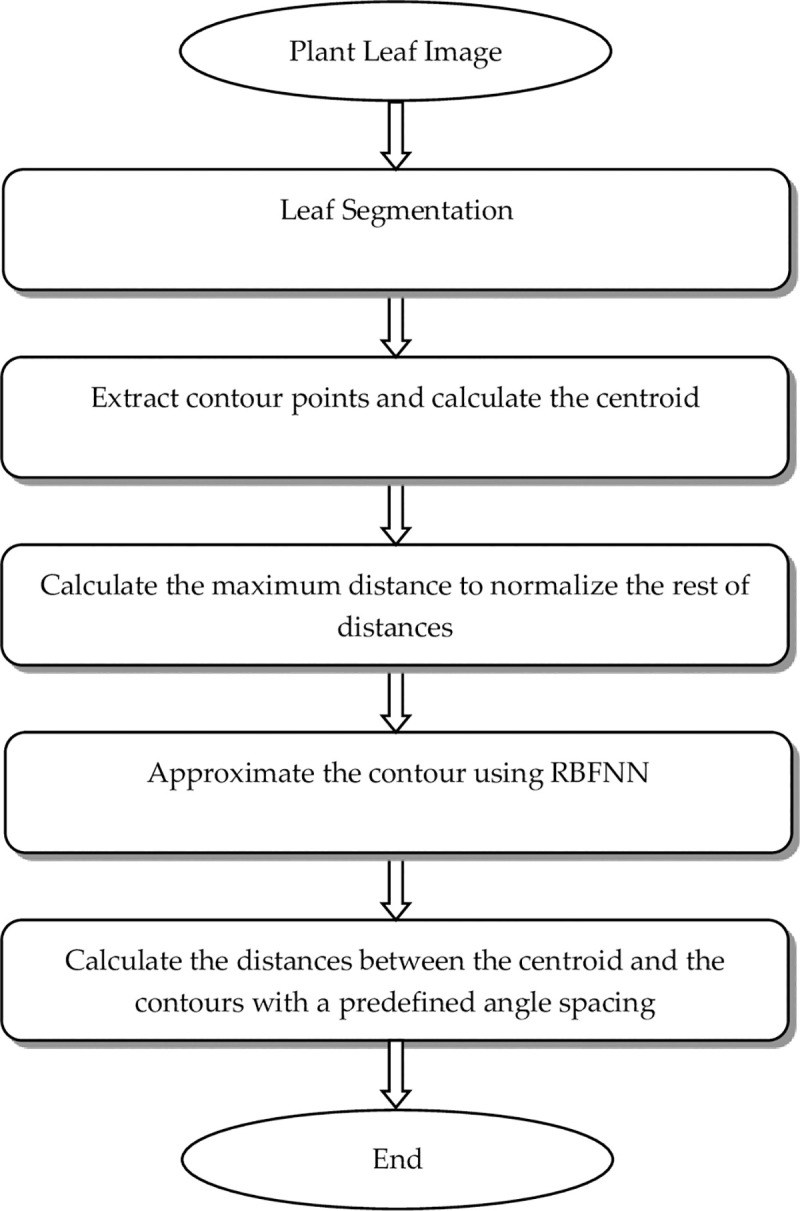
The proposed feature extraction algorithm.

The proposed feature extraction algorithm uses the plant leaf shape, as illustrated in Fig 2, and is explained in detail as follows.

### A) Image shape signatures

Shape signature is one of the most commonly used approaches to represent contour descriptors. It represents the shape using a one-dimensional function calculated from the contour points of the leaf's shape. There are plenty of shape signatures used in the literature. One of the most frequently used shape signatures is the centroid contour distance (CCD) according to [27, 28, 29, 30]. The studies in [31, 32, 33], calculated centroid contour distance plot and angle code histogram (ACH) for leaf representation. The authors in [34] represented the leaf shape using centroid contour distance curve and proposed an ontology-based integrated approach for classification. The extracted features points from the boundary are then stored in an ordered data structure such as linked list. Invariant moments are used to represent leaf’s shape.

The centroid of a discrete-based shape is computed using the following equations:

$$X_{Cent}=\frac{1}{N}\sum_{i=1}^{N}X(i)\;and$$ (14)

$$Y_{Cent}=\frac{1}{N}\sum_{i=1}^{N}Y(i)$$ (15)

Where N is referring to the number of image’s boundary points. If we define the coordinate set of contour points of the shape as:

(x(i),y(i)),i=1,2,…,N,

By considering the centroid of the leaf shape as a central reference point, the distance *D*_*c*_ between the centroid and each feature point can be calculated using the following equation.

$$D_{c}=\sqrt{{\left(x(i)-X_{Cent}\right)}^{2}+({y(i)-Y_{Cent})}^{2}},i=1,2,\ldots ,N$$ (16)

To make the computed distances scaling-invariant, they were divided by the maximum distance and stored in a circular list.

RBFNNs are used in this stage for regression. They are applied as a curve fitting technique on the dataset to get the leaf contour. In which RBFNN parameters were adjusted to find the continuous relation between angle and contour distance using GA optimization. Those parameters include the total number of units in the hidden layer, RBF centers and standard deviations to minimize the RMSE below 1% for the testing data. The maximum number of generations for GA was set to 1000.

The dataset for each number of features were randomly arranged. The features representing the dataset are divided into training and testing datasets. One way to evaluate the classifier model is to split the training dataset into training and validation datasets, then train the models against the smaller training dataset and evaluate them against the validation dataset using k-fold cross-validation. We randomly split the training dataset into 10 distinct subsets called folds, followed by training and evaluating the classifier model 10 times, using a different fold for evaluation every time and training on the other 9 folds. The result is an array that contains the 10 evaluation scores. Moreover, cross-validation allows to get not only an estimate of the performance of the model, but also a measure of how precise this estimate is. As the dataset was medium-sized, 80% of the dataset features were used for training and validation, while 20% of the dataset were used for testing.

### B) CCD sequence from shape boundaries

The information is captured from the shape’ boundary using the description of shape sequences. The appropriate formulation of centroid contour distances using RBFNN curve fitting as well as its properties according to scale, translation and rotation invariance is discussed. The centroid contour distance (CCD) sequence represents the distance between each contour boundary point and the contour centroid. One of the characteristics of CCD curve sequence is its translation invariance as well as scale invariance under certain normalization.

**Fig 3:**
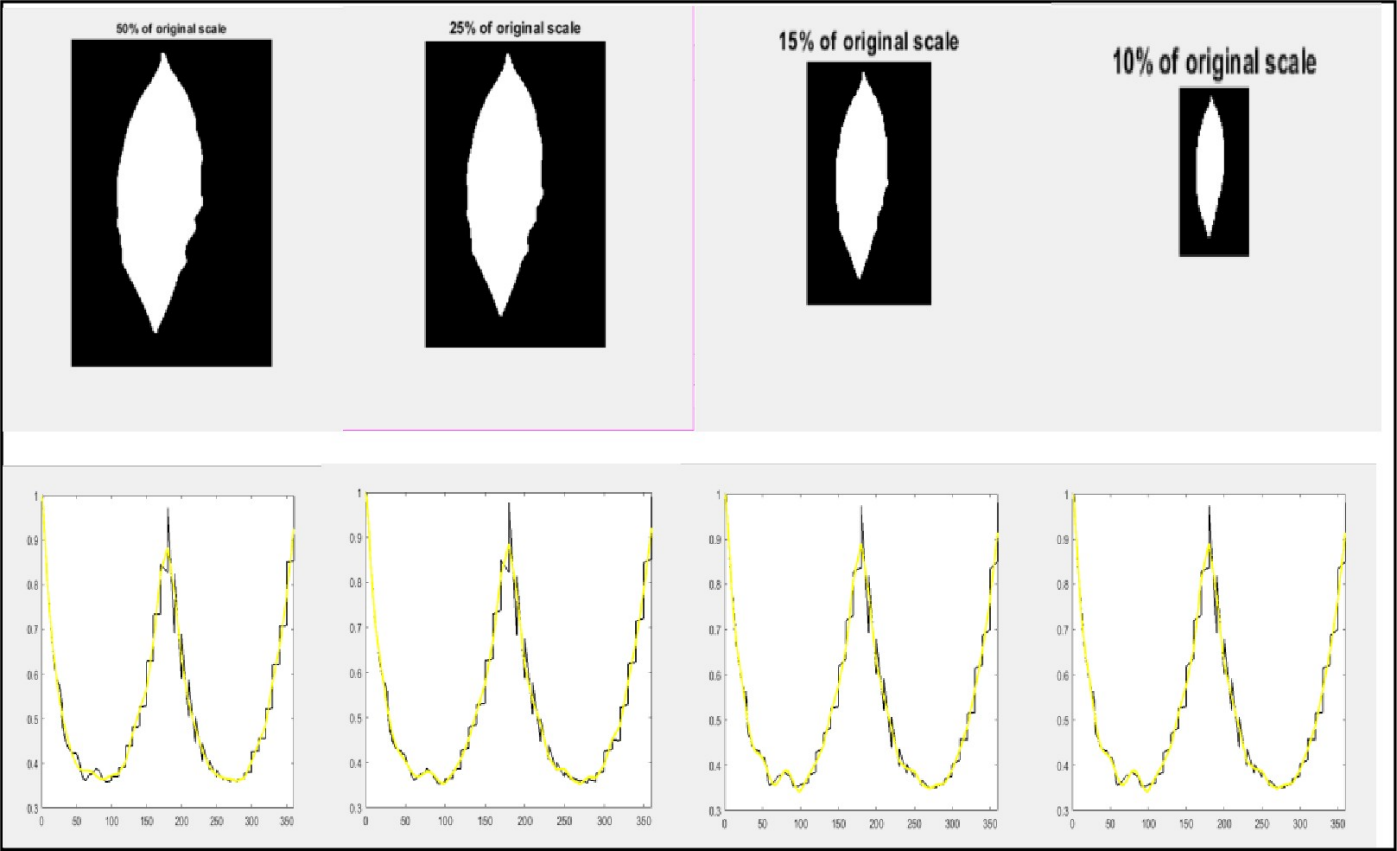
**A binary leaf image reduced to different sizes and their corresponding curve-fitted lines to verify scale invariance.** The horizontal axis of curve-fitted lines represents the angle’ values and the vertical axis of the curve-fitted lines, represent their corresponding normalized CCD values.

**Fig 4:**
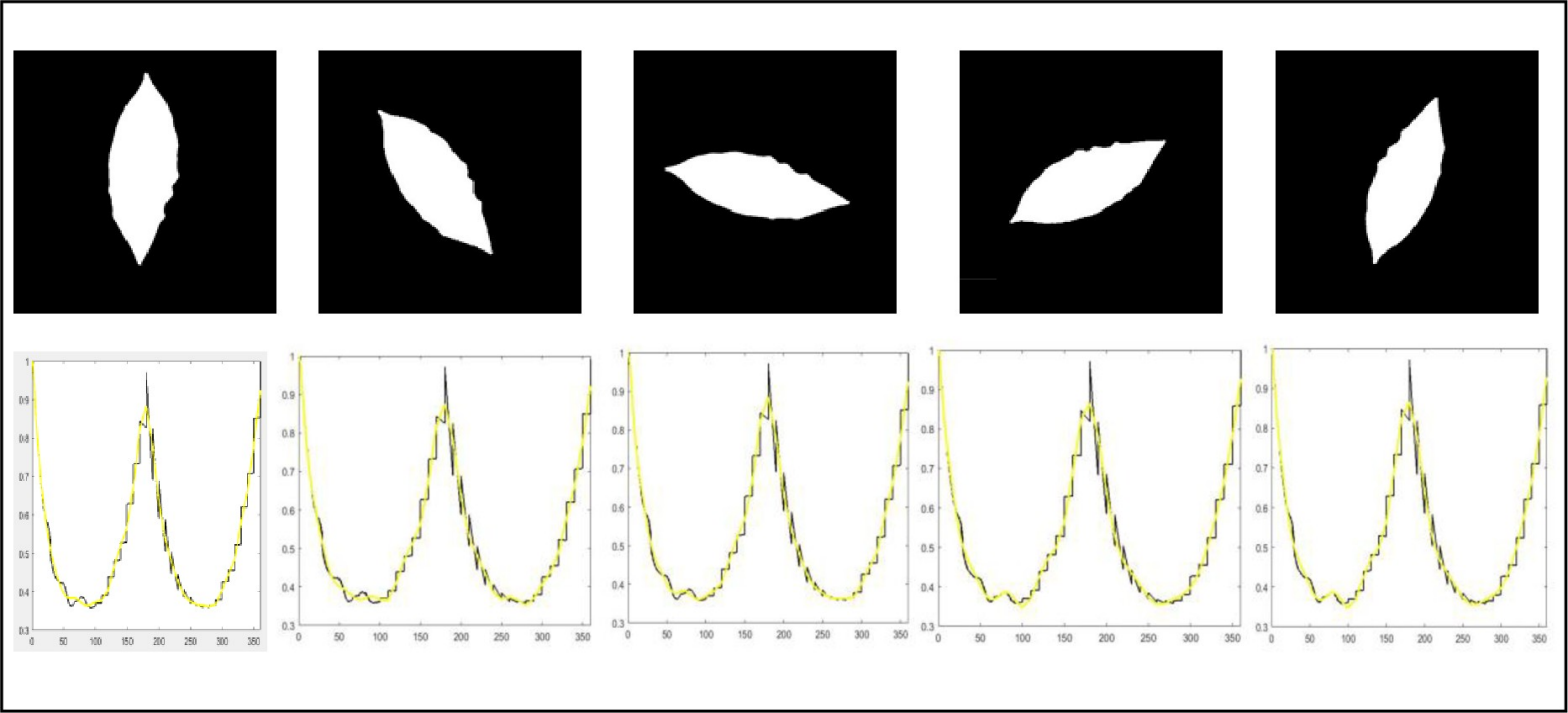
Binary leaf image rotated anti-clock wise from the origin degree to 40^*o*^, 80^*o*^, 120^*o*^, and 160^*o*^ from left to right respectively to verify rotation invariance with their corresponding curve-fitted data. The horizontal axis of the curve-fitted lines represents the angle values, and the vertical axis of the curve-fitted lines represents the corresponding normalized CCD values.

**Fig 5:**
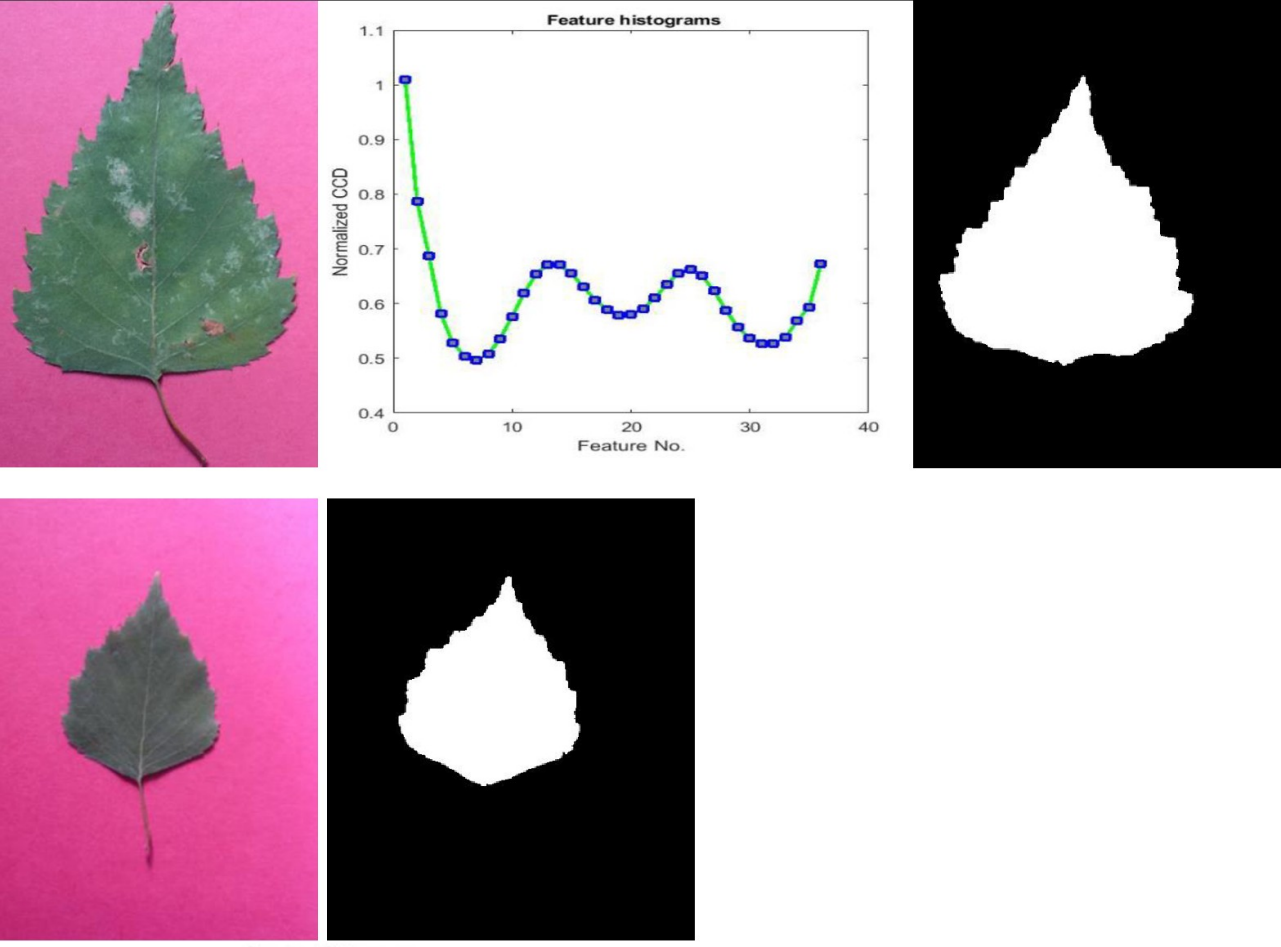
Two different leaf images belonging to the same plant species and their corresponding contour feature curves.

To prove the scale invariance of the proposed scheme, a binary leaf image was reduced to different sizes. Then, the corresponding curve fitting is created using the features of these images as shown in Fig 3. The features used are the normalized distances as measured relative to the maximum distance between the centroid and the farthest point on the contour. Therefore, the scale effect will vanish by the normalization process. Therefore, the normalized features are scale invariant. Also, to prove the rotation invariance, a binary leaf image was rotated anti-clockwise to different degrees and then their features were extracted from the rotated images to compute their corresponding curve fitting as shown in Fig 4. Because the features depend on the maximum distance between the centroid and the farthest point on the contour as the reference axis, that means features are rotation invariant as the reference axis will be the same in any rotated leaf. An example of declaring that different plant leaves be members of the same species may contain great differences in their shape contour as shown in Fig 5. This figure shows that two plant leaves belong to the same species have different feature curves.

## IV. Results and discussion

This section evaluates the performance of recognizing the various plant species. The utilized classification techniques, namely RBFNN, SVM and an optimized version of the SVM using a metaheuristic optimization technique known as Salp Swam Algorithm will be applied and compared using the proposed leaf features extraction.

### A) Dataset

In this research, we used plant leaves dataset that publicly available from UCI [35, 36]. The dataset contains leaves images for about 100 plant species. Each one includes sixteen data shapes. Each data shape is characterized by 64 features vectors obtained from the normalized contour signatures. These signatures were calculated from the differentiation of shape image’s foreground and background pixel.

### B) RBFNN based contour fitting

**Fig 6:**
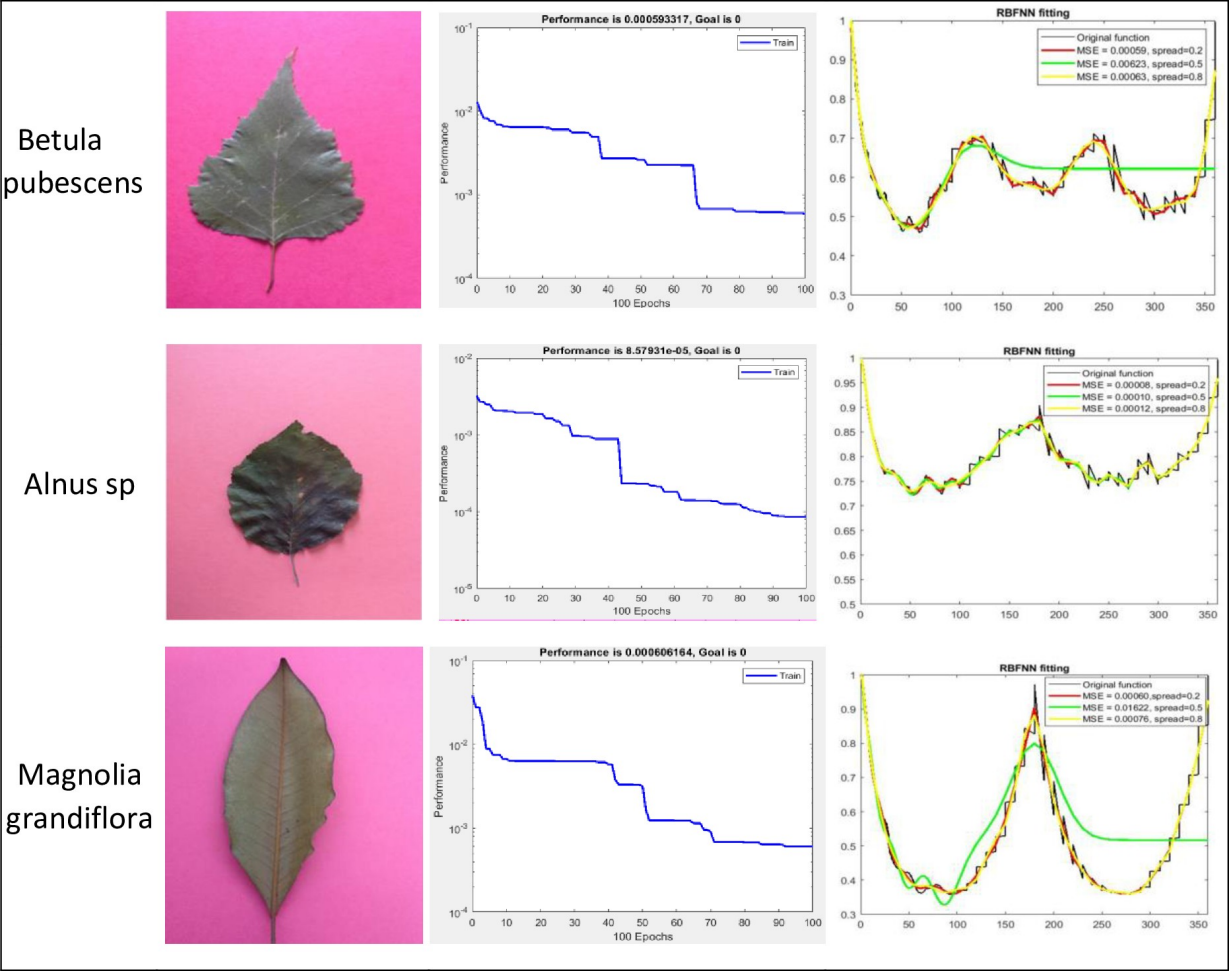
Different leaf species with the corresponding Mean Square Error (MSE) and RBFNN curve fitting. RBFNN curve fitting.

**Fig 7:**
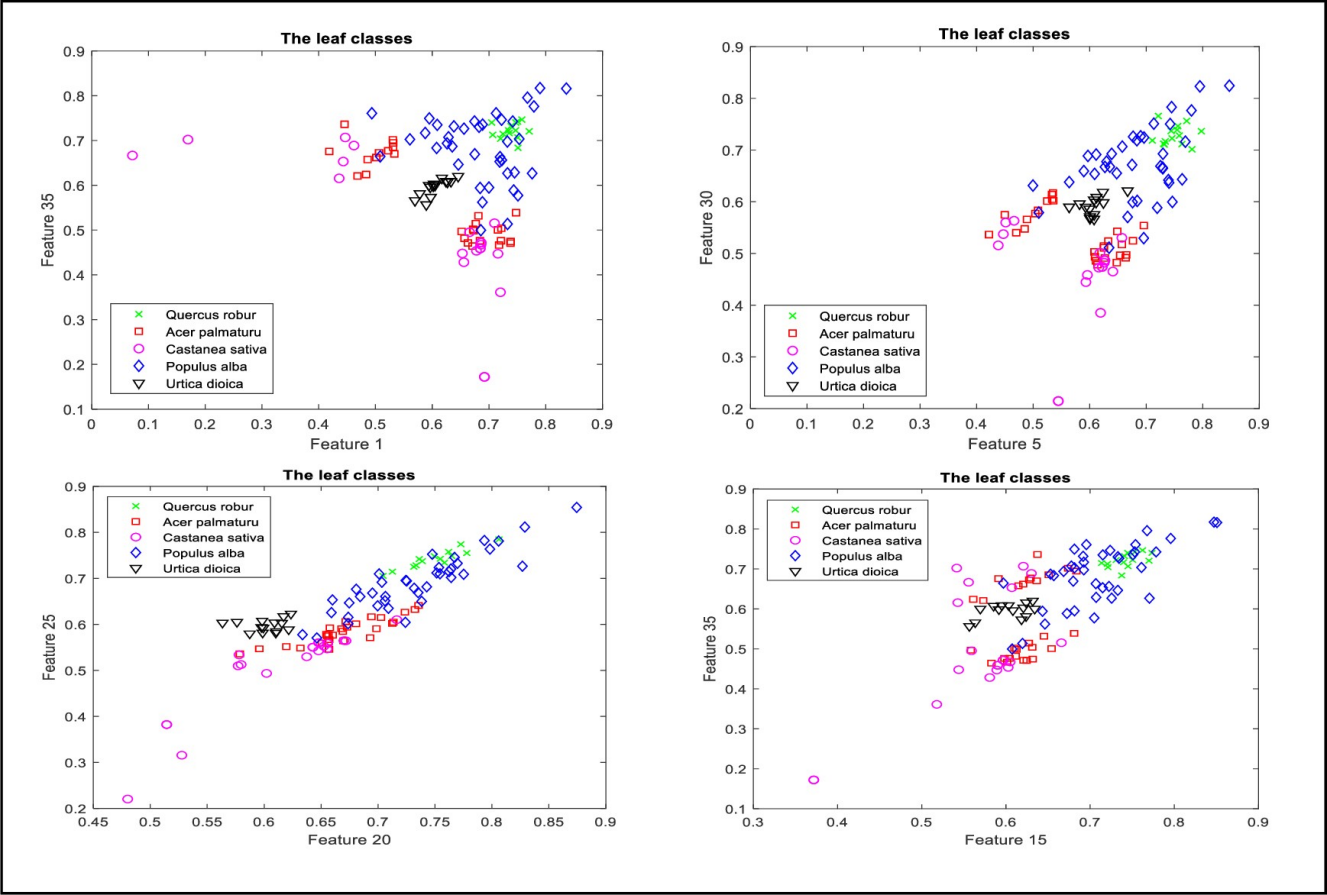
Pairs of features from the 36 features defining the leaf and their corresponding leaf classes.

The centroid for the contour points for each leaf was calculated, and the contour points’ distances to that centroid were normalized relative to the maximum distance. Those normalized distances represented the dependent variable while the angles of those lines were calculated relative to the maximum contour distance to the centroid as the independent variable. RBFNN was then used to find the best curve that minimizes the RMSE for each leaf in the dataset. The performance of that curve fitting is shown in Fig 6 in which several leaf examples were illustrated. For the RBFNN, the optimized curves along with other curves with different spread values, were shown to demonstrate the effect of the parameter on the fitting accuracy. Fig 7 illustrates the different classes plotted according to different pairs of features. The graphs in two dimensions show the complexity of separating the leaf’s classes and the necessity for the classifiers to perform well in the complex and high dimensional problems.

### C) RBFNN classifier

The number of features points for each leaf were selected to be of different sizes. Those features are uniformly distributed starting from angle 0^*o*^ relative to the maximum distance between the contour and the centroid and is incremented by a constant angle to achieve the required number of features. Those same features will be used by the other two classifiers to ultimately choose the number of features that corresponds to the best performance. The dataset features were arranged randomly while 80% of the dataset were used for testing and validation using 10-fold cross-validation while 20% were used for testing. The choice of the ratio of 80:20 for training and testing was done because of the medium-sized dataset.

The Genetic algorithm was used here to tune the RBFNN parameters (weights, *μ*, and *β*) to achieve minimum RMSE with a population size of 20 solutions and 1000 number of generations.

### D) SVM classifier

**Fig 8:**
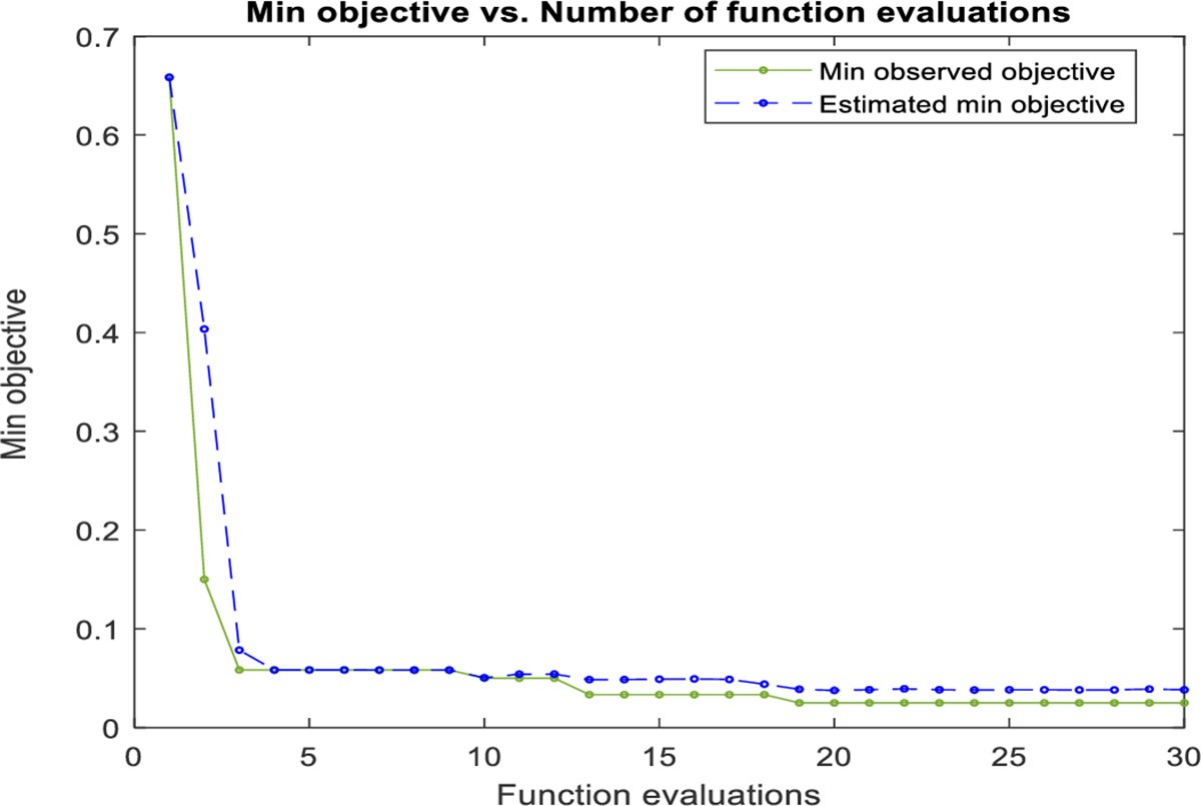
The SVM objective function minimization against iterations.

**Fig 9:**
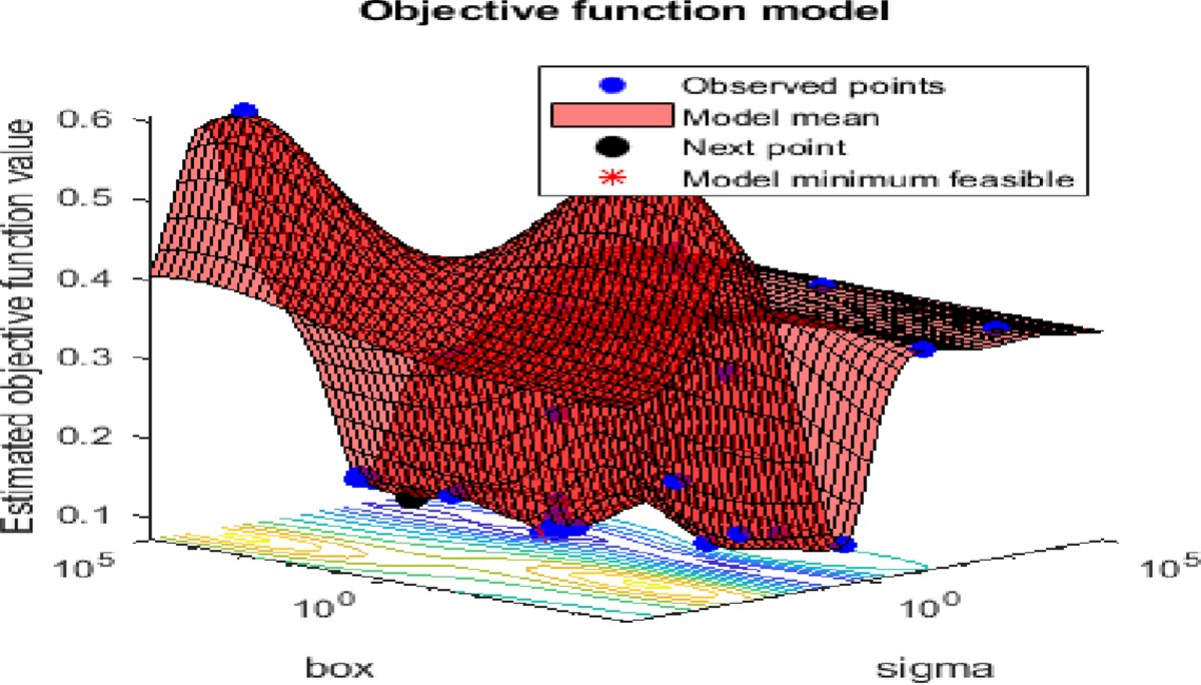
The SVM kernel parameters optimization using Bayesian optimization.

SVM classifier was applied on the dataset using the Gaussian kernel. However, the minor increase of the value of *σ*, will increase the fitting for the training data on the expense of the generalization error and can cause over-fitting problem. While the regularization parameter *C* compromises between smooth decision boundary and correctly classifying the training points. Therefore, to achieve better classification accuracy, these parameters were optimized by minimizing the mis-classification rate on the datasets and accordingly ensure high classification performance for the leaf dataset using Bayesian optimization approach. The training curve shown in Fig 8 illustrates the steep descent of the objective function value with the number of iterations (function evaluation). While Fig 9 shows the kernel parameters optimization using Bayesian optimization technique. It is worth mentioning here that SVM is very similar to RBFNN and can achieve comparable results. However, the excessive number of parameters used by RBFNN can be reflected in the algorithm complexity, and that can, in turn, lead sometimes to a lower performance if the parameter selection is not optimized.

### E) SSA based SVM classifier

The salps’ positions are first initialized using random numbers. The searching limit of the parameter *C* of the support vector machine (SVM) was limited by *C*_*min*_ = 0.01 and *C*_*max*_ = 1000, and the searching limit of σ was determined by *σ*_*min*_ = 0.01 *σ*_*max*_ = 100. If these limits increase, it will enlarge the search space; and accordingly, more salps will need to be utilized to search for the optimal solution and that will lead to more computation and consequently a slow convergence rate.

**Fig 10:**
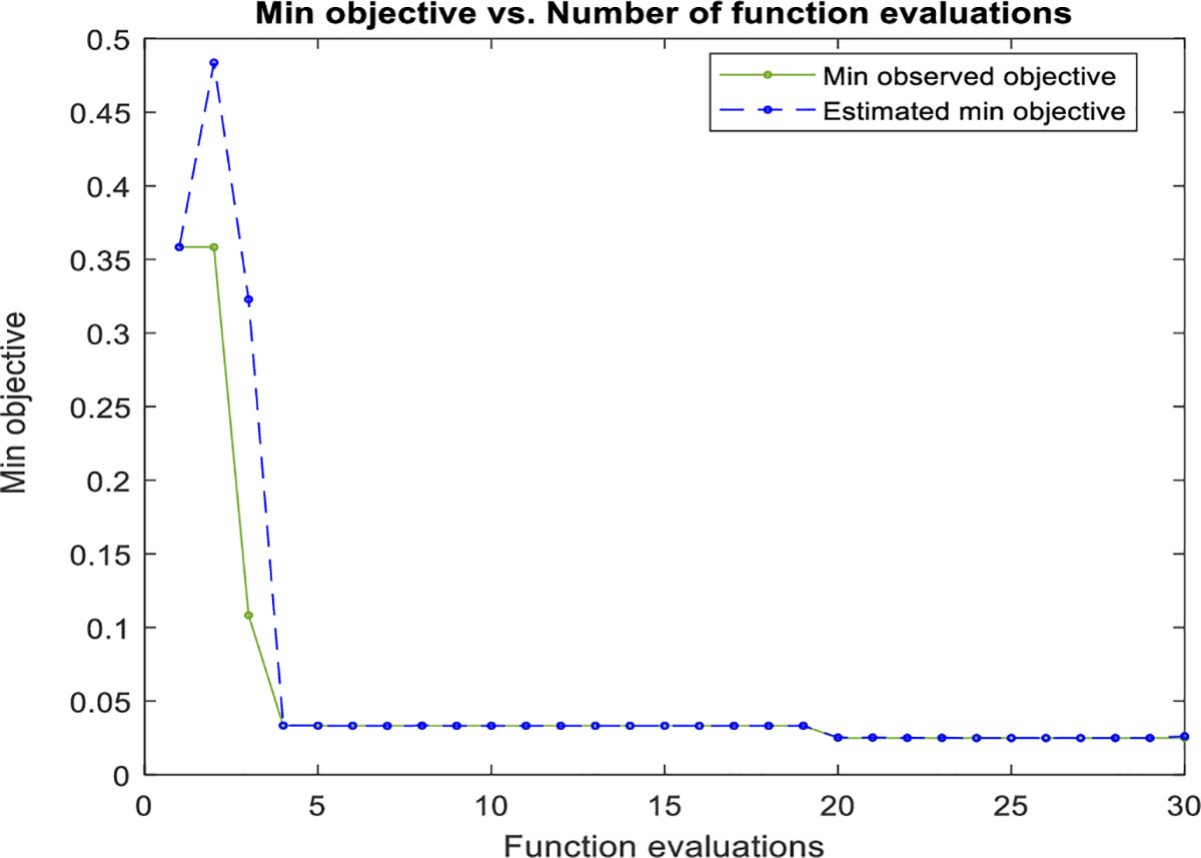
The SSA based SVM objective function minimization against iterations.

**Fig 11:**
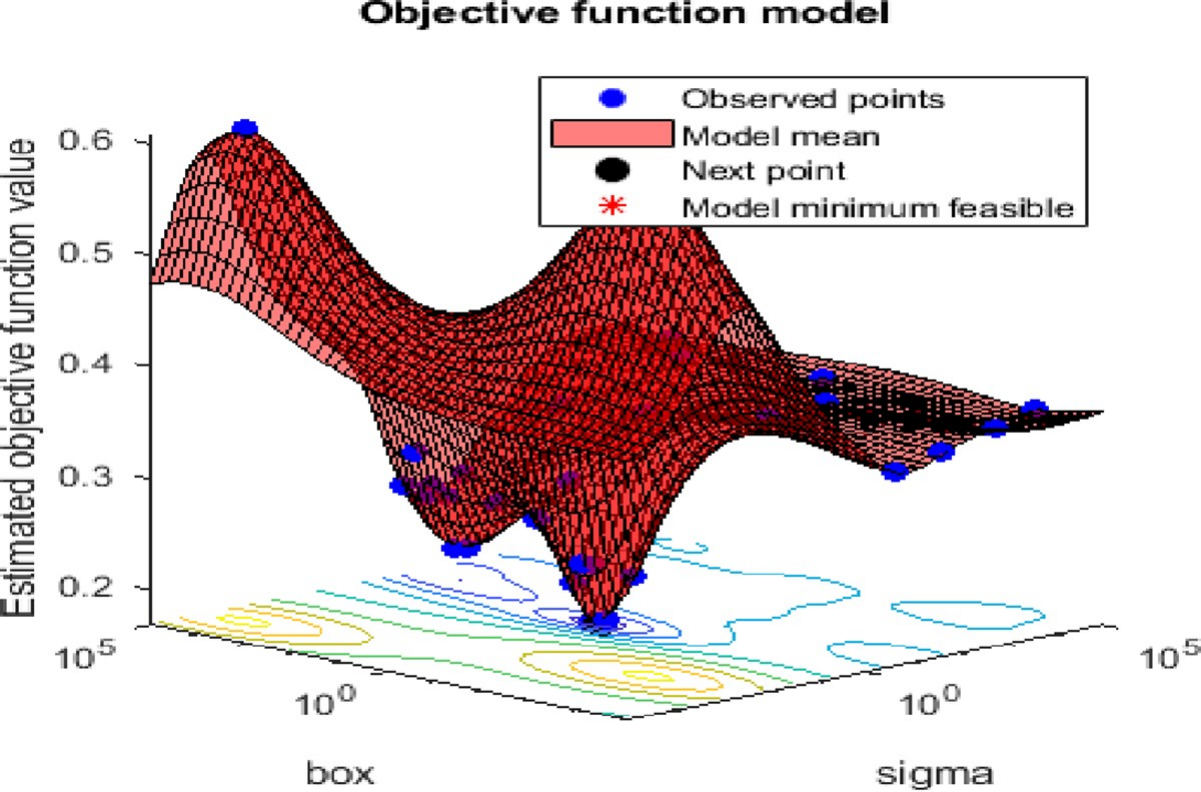
The SVM kernel parameters optimization using SSA optimization.

In this research, the number of search agents is 20, while the algorithm is iterated until the objective function value of 0.025 is met. Fig 10 shows the performance curve (minimum objective function achieved) for the algorithm plotted against the iterations during the training stage. While Fig 11 shows the kernel parameter optimization using the SSA optimization technique. The SSA based SVM gives a slightly faster curve to reach the objective function than in the conventional SVM as shown in Fig 8.

In order to find the best features, different angles were selected relative to the leaves reference axes. The experiments started with an angle of 30^o^ which corresponds to 12 points on the contour representing the leaf’s features. The three proposed classifiers have been applied using the dataset of all the leaves’ features and the classification accuracies were calculated. The experiments were then repeated for angles 20^o^, 10^o^, and 8^o^ which correspond to numbers of features 18, 36, and 45 respectively. The results achieved by the three classifiers are summarized in Table 2.

**Table 2:** The proposed system accuracies with respect to different selected angles (number of features).

Method	Angle = 8° (45 features selected)	Angle = 10° (36 features selected)	Angle = 20° (18 features selected)	Angle = 30° (12 features selected)
RBFNN	90.71%	91.45%	90.41%	89.95%
SVM	92.83%	93.33%	91.04%	89.14%
SSA based SVM	95.52%	96.67%	92.03%	90.15%

As inferred from Table 2, the best performance of the proposed classifiers has resulted from using a 36 features dataset. Therefore, the three corresponding classifiers were further evaluated. Performance metrics comparison is summarized in Table 3, in which the processing time for each technique was evaluated and broke down into the different task times consumed during the training and validation stage. In all the classification techniques evaluated in this research, k-fold cross-validation was used in which data were partitioned to nearly k subsets with the same approximate sizes. The platform used in this research to develop the three classification techniques is implemented using a PC with Intel(R) core (TM) i7-7500 CPU, 2.7 GHz, 16 GB RAM, Windows 10 operating system, and MATLAB (R2019b).

**Table 3:** Classification parameters of the proposed three classifiers.

Factor	RBFNN Classifier	SVM Classifier	SSA based SVM Classifier
Total elapsed time (s)	167.3542	65.5933	100.9488
Total objective function evaluation time (s)	114.2672	31.4298	68.6173
Function evaluation time (s)	0.21056	0.20742	0.19765
Objective function value	0.02404	0.02437	0.02389
Classification performance	91.45%	93.33	96.67

The relative performance of consumed time and classification accuracies showed that RBFNN consumes more time to reach the objective function value (0.025) and at the same time with a classification accuracy of 91.45%. While SVM classifier managed to achieve the shortest consumed time with a relatively high classification accuracy of 93.33%, which can compare well with the other findings in the literature. On the other hand, SSA based SVM managed to achieve a high classification accuracy of 96.67% on the expenses of slightly longer time consumption. Therefore, the SSA based SVM combined with the RBFNN feature extractor based on the shape of the leaf could offer a promising paradigm in similar applications.

## V. Conclusions

RBFNN is widely used in complex applications with a great accuracy in both regression and classification. It has been used here to find the best fitting curve for each leaf contour data points to allow for any selection scenario of features. That can be important in similar problems that rely on the object shape and is able to avoid the effect of rotation, scaling, and translation. The RBFNN was used again in the classification stage with considerable classification accuracy compared to the other methods found in the literature. On the other hand, when SVM was used with the selected features, an accuracy of 93% was reached, which outperformed the RBFNN classifier. The parameters of the RBF kernel used in the SVM classifier were the key for further SVM accuracy improvement. Therefore, SSA, a recent optimization technique that belongs to the meta-heuristic techniques that proved to be superior in avoiding local optima due to their stochastic nature, was considered. It was modified in a way to be slower when it is too close to the optimal point. That was done due to the fact it is a quasi-optimal technique that never guarantees the solution reached to be the optimal one. When the SSA was used with SVM, it reached 96.67, which represent a significant performance increase compared to the RBFNN classifier and the SVM classifier. That shows the potential in many similar fields that might benefit greatly from a features’ extractor using RBFNN and a classifier using the SSA based SVM. Moreover, a future research direction is to investigate other applications that incorporate geometric features in image processing, especially those with irregular shapes, which can elaborate on the proposed feature extraction approach.
